# Supplementary material for: Skeletal Muscle-Derived Stem Cell Transplantation Accelerates the Recovery of Peripheral Nerve Gap Injury under 50% and 100% Allogeneic Compatibility with the Swine Leucocyte Antigen
Source: Biomolecules. 2024 Aug 2;14(8):939. doi: 10.3390/biom14080939 (PMC11353188; doi:10.3390/biom14080939)
Supplement: Supplementary file 1 [file biomolecules-14-00939-s001.zip › Figure S1.pdf]

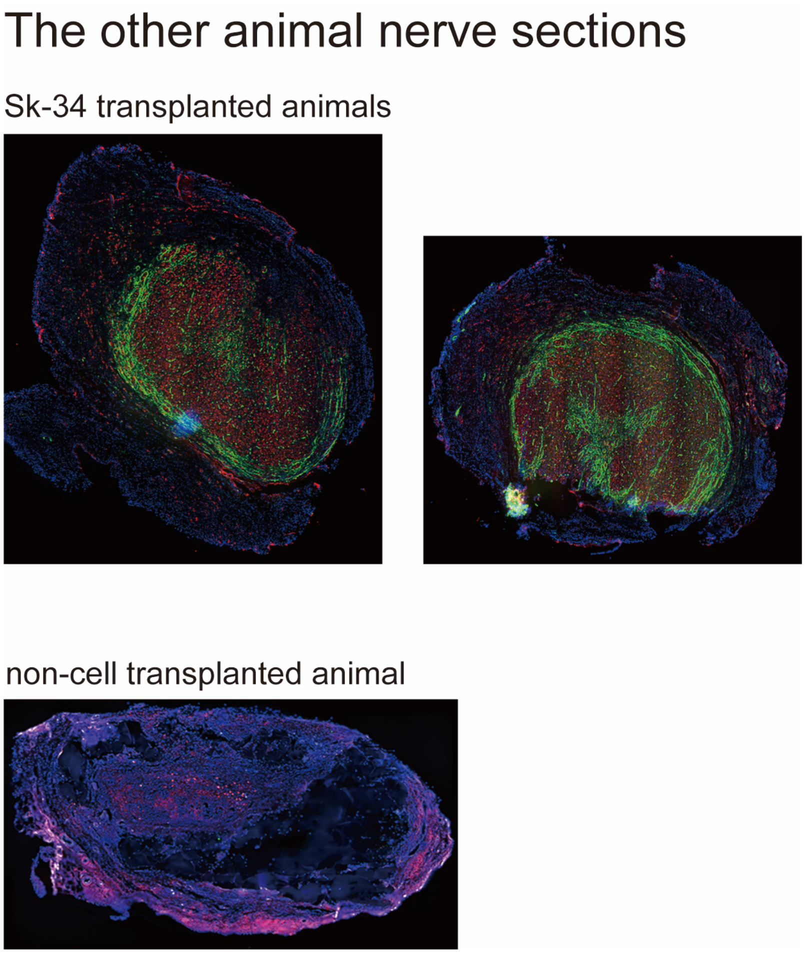

Figure S1: Additional morphological evidence of recovery in other animals.
